# Supplementary material for: Recrystallization of Si Nanoparticles in Presence of Chalcogens: Improved Electrical and Optical Properties
Source: Materials (Basel). 2022 Dec 11;15(24):8842. doi: 10.3390/ma15248842 (PMC9787536; doi:10.3390/ma15248842)
Supplement: Supplementary file 1 [file materials-15-08842-s001.zip › materials-2081978-supplementary.pdf]

Supplementary Materials

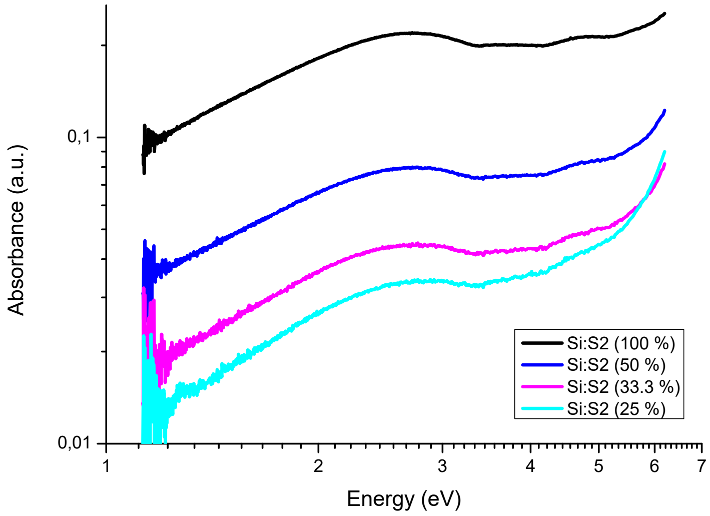

Figure S1. Absorbance spectra of Si:S2 at various dilutions

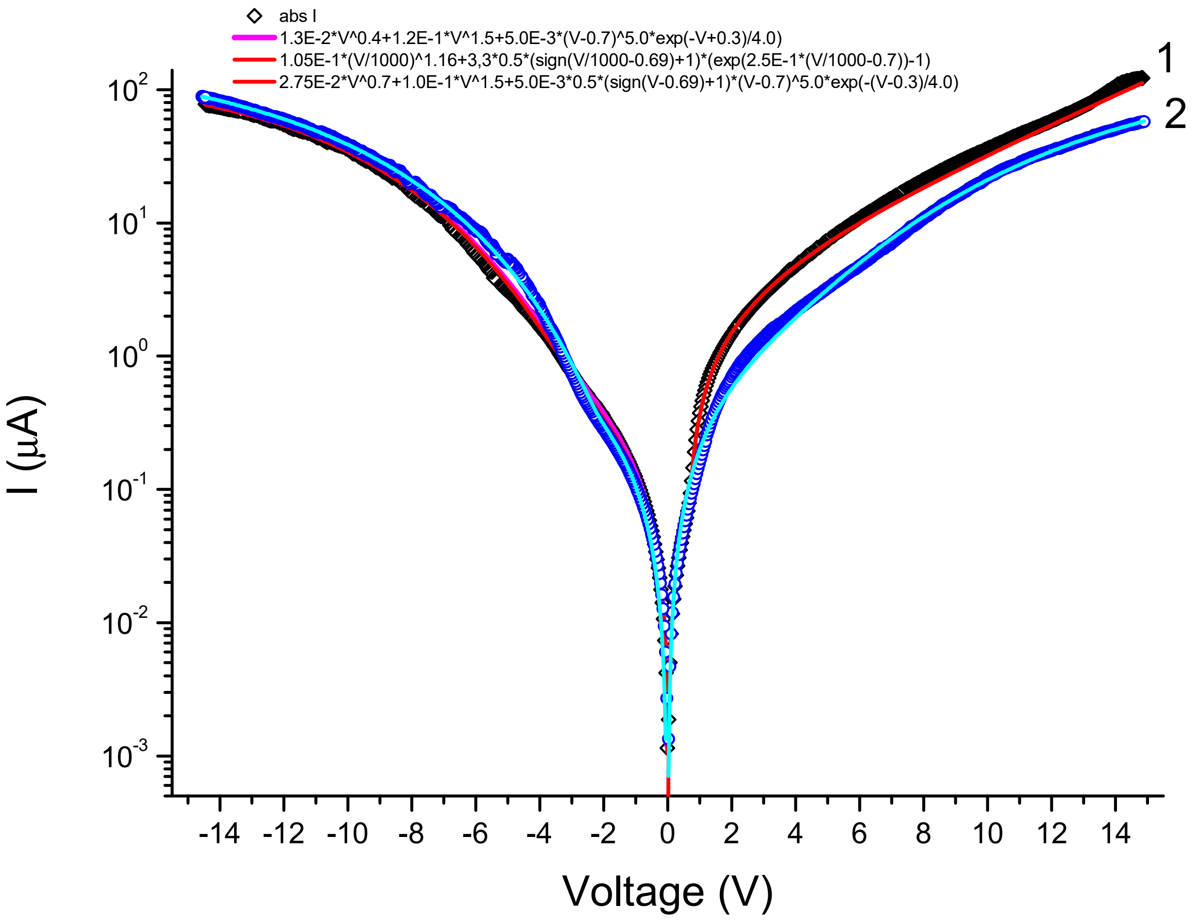

Figure S2. I-V characteristics of Si:S5, obtained at room temperature: (**1**) at pressure of surrounding air atmosphere of $3 \cdot 10^{-4}$ Torr. Solid lines mean numeric approximations with following functions: $I_{forw} = I_{01} \cdot V^{1.16} + I_{02} \cdot e^{V/A}$ for forward current and $I_{rev} = I_{01} \cdot V^{1.5} + I_{02} \cdot (V - V_0) \cdot \exp(-(V - \alpha)/\beta)$ for reverse current. (**2**) At

atmospheric pressure after 51 minutes of sample being in contact with air. Solid lines mean numeric approximations with following functions: $I_{forw} = I_{01} \cdot V^{1.5} + I_{02} \cdot V^{5.5} \cdot \exp(-V/\eta)$ for forward current and $I_{rev} = I_{01} \cdot V^{1.5} + I_{02} \cdot V^{2} + I_{03} \cdot (V - V_0)^{5} \exp(-V/\varkappa)$ for reverse current.

.

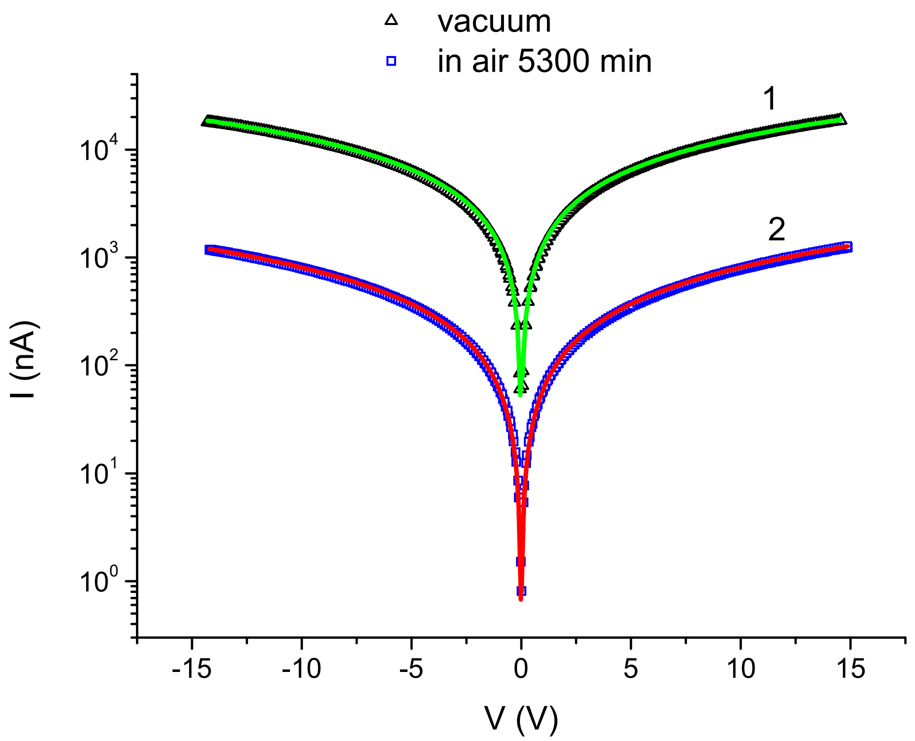

Figure S3. I-V characteristics of Si:Se film measured at room temperature. (**1**) Measured in vacuum; (**2**) Measured at atmospheric pressure after 5300 minutes of sample being in contact with air. Solid lines mean numeric approximations with following functions: I = 1300·V for the green curve and $I = 60 \cdot V^{1.13}$ for the red curve.

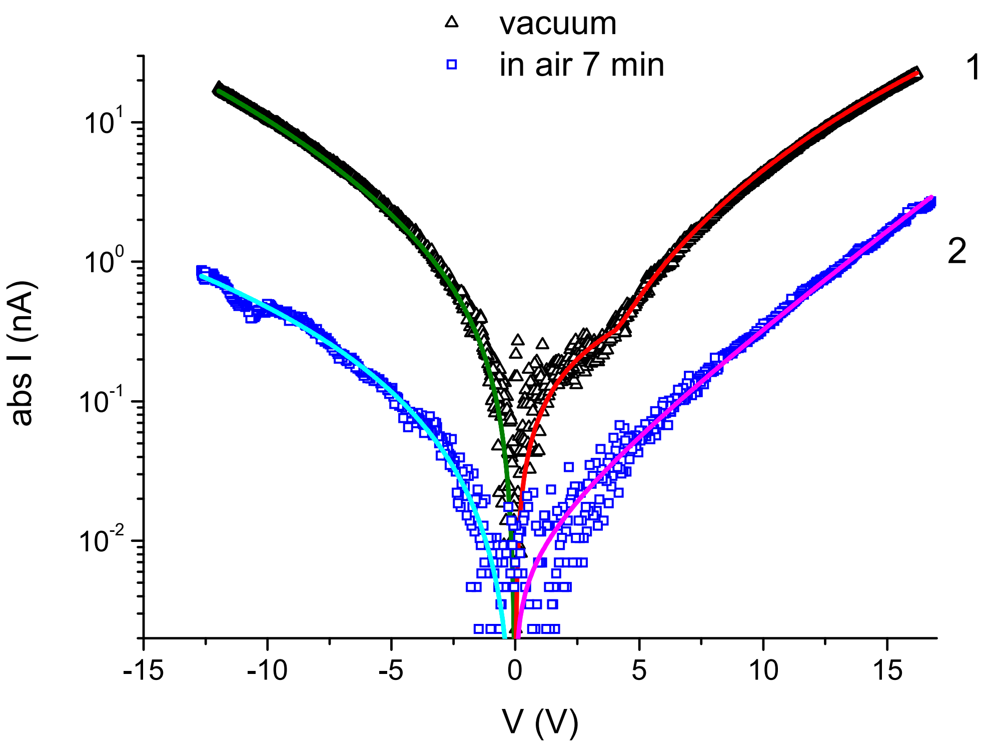

Figure S4. I-V characteristics of Si:Te film measured at room temperature. (**1**) Measured in vacuum; (**2**) Measured at atmospheric pressure after 7 minutes of sample being in contact with air. Solid lines mean numeric approximations with following functions:

for forward current: $I_{forw} = 0.08 \cdot V +$ (0.15 for V>4, otherwise 0)$\cdot(V-4)^{1.5}$ + (0.005 for V>4, otherwise 0)$\cdot(V-4)^{3.2}$;

for reverse current: $I_{rev} = -0.15 \cdot (-V)^{1.5} - 0.0017 \cdot (-V)^{3.5}$ After 7 minutes of exposure to air: $I_{forv} = 0.0059 \cdot V^{0.5} \cdot \exp(V/3.5)$, $I_{rev} = 0.007 \cdot (-V)^{1.5} + 0.0005 \cdot (-V)^{2.7}$.
